# State of rheumatic fever in Algeria. Viewpoint of a cardiac surgeon.

**DOI:** 10.1186/1749-8090-10-S1-A3

**Published:** 2015-12-16

**Authors:** Abdelmalek Bouzid, Salim Chibane, Mohamed Atbi, Halima Larbi, Boukri Hamouda, Redha Djilali-Sayeh, Youcef Larabi, Tarek Hamdi, Sami Bouchenafa, Ramdan A Ould Abderrahmane

**Affiliations:** 1 Department of Cardiac Surgery, EHU 1er Novembre 54, Oran 31000, Algeria

## Background/Introduction

In Algeria prevalence of rheumatic fever(RF) has steadily decrease especially after the establishment of the national program against the RF in 1990; the national incidence decreased from 04.7 /100,000 in 2002 to 02.5 /100,000 in 2003, 02.3 /100,000 in 2004, in 2009 the national incidence decreased to 1.01cas /100,000 of 04 to 19 years.

However, the share of rheumatic patients requiring surgical treatment for valvular lesions has not changed, indicating the insufficiency of the modified Jones criteria.

## Aims/Objectives

Our aim is to evaluate the sensitivity of the Jones criteria for the diagnosis of rheumatic fever in patients with rheumatic valve disease.

## Method

77 patients were operated for pure rheumatic mitral stenosis, from January 2009 to May 2012 at the cardiac surgery department of the EHU 1 November 54.

The sex ratio was 0.33; the average age was 42.26 years 95% [39.84-44.68].

Rheumatic lesion was confirmed by pure stenotic lesion of the mitral valve.

## Results

Only one patient of the 77 patients (1.3%) had a history of rheumatic fever diagnosed and treated; the remaining 76 patients (98.7%) who have never been diagnosed or treated for rheumatic fever, until the appearance of valvular lesions which indicated surgery.

## Discussion/Conclusion

Despite the favorable results of the national program against the RF; However cardiac surgery departments are still receiving patients with valvular rheumatic lesions, which have never been diagnosed or treated, this testifies to the insufficiency of Jones criteria for the diagnosis of rheumatic fever, other criteria must be introduced for the diagnosis (echocardiography, biological); the aim being to reduce the socio-economic impact of this disease, and why not eradicate definitively rheumatic fever.

